# Transition to recycling versus incineration in municipal solid waste management: Evaluating the speed of greenhouse gas emission reduction

**DOI:** 10.1177/0734242X251340318

**Published:** 2025-08-04

**Authors:** Anna Zaikova, Amirmehrab Falsafi, Amirsohrab Falsafi, Mariam Abdulkareem, Mika Horttanainen

**Affiliations:** 1 Sustainability Science, Lappeenranta-Lahti University of Technology LUT, Lappeenranta, Finland; 2 Natural Resources Institute (Luke), Helsinki, Finland

**Keywords:** Landfill diversion, municipal solid waste, global warming potential, material recovery, waste-to-energy, speed, marginal data

## Abstract

With the urgent need for climate action, reducing landfill emissions is crucial. Motivated by potential differences in time required to transition from landfilling to recycling versus incineration of municipal solid waste (MSW), this study assesses which of the two pathways offers a quicker and more robust solution for mitigating greenhouse gas (GHG) emissions in MSW management. The speed of transition to waste incineration and recycling was analysed retrospectively among frontrunners in landfill reduction. In three selected cases, the speed of GHG emission reduction resulting from their transition mainly to waste incineration (Finland), recycling (Italy) and a combination of those (Poland) was calculated using life cycle assessment (LCA). Robustness of the results was tested by varying key parameters. The findings show that GHG emission reduction and its speed depend significantly on the energy sources being replaced on the market by energy derived from waste. Depending on trends in the energy market and how they are reflected in the LCA model, the effects of transitioning to incineration range from the largest and quickest GHG emission reductions to no environmental benefit. Taking into account the energy sector transition projections for various countries, potential cases were identified where transitioning to incineration can reduce GHG emissions faster than recycling. However, significant uncertainty arising from assumptions about the energy replaced, which was present even in retrospective analysis, highlights the need for robust solutions for emission reduction in future MSW management – solutions that can only be developed by comprehensively accounting for these uncertainties.

## Introduction

Many countries promote diverting municipal solid waste (MSW) from landfills to more efficient waste management practices, such as material recovery and waste incineration. Despite the progress in landfill reduction in Europe, global waste management practices lag, with a significant reliance on landfilling due to its cost efficiency (Kaza et al., 2018). Landfills continue to produce emissions through leaching and possible landfill fires (Vassiliadou et al., 2009) as well as formation of greenhouse gases (GHGs; Siddiqua et al., 2022). Landfill gas (LFG) that is formed as a result of decomposition of organic waste contains significant amounts of methane, a potent GHG. Global warming potential (GWP) of methane is 28–36 times higher compared to carbon dioxide when considering its impact over a 100-year period (IPCC, 2006). Reduction of methane emissions is an important objective in combatting climate change, with a target set for 2030 (European Commission, 2020). The IPCC (2022) emphasized the urgent need for reduction of GHG emissions in general.

Reduction of methane emissions from landfilling is typically addressed by recycling and municipal solid waste incineration (MSWI) – in this article, the latter refers specifically to incineration with energy recovery. Both recycling and MSWI are generally considered to have a lower environmental impact than landfilling and contribute to GHG emission reduction, as shown by various case studies (e.g. Calabrò, 2009; Chen et al., 2022; Malinauskaite et al., 2017; Richard et al., 2021; Schneider et al., 2012; Verma and Borongan, 2022). In recycling, the GHG emission reduction can result from lower amount of energy required to produce secondary materials, when compared to primary material production, with recycling of aluminium being a prime example (Al-Alimi et al., 2024). Energy recovered in MSWI reduces the need for conventional energy sources and thus allows to avoid GHG emissions associated with conventional energy production. Moreover, when MSW is recycled or incinerated instead of landfilling, methane emissions are not generated, as waste decomposition does not occur.

A transition from landfilling to MSWI or recycling requires significant changes in the waste management infrastructure and operations. Building MSWI plants requires large capital investments (Xin-Gang et al., 2016), but can be implemented relatively quickly, taking approximately 5–8 years from planning phase to operation of an MSWI plant. Meanwhile, developing a recycling system and reaching sufficient level of recycling can take longer time, as recycling performance is dependent on many external factors such as involvement of citizens, the quality of collected recyclables, market of secondary materials, etc. (Gundupalli et al., 2017; Stoeva and Alriksson, 2017; Zhang et al., 2017). Therefore, the transition to MSWI and recycling may lead to different speeds of GHG emission reduction, which is critical given the urgent need to mitigate climate impacts. However, existing research has not examined potential emission reduction when transitioning to recycling and MSWI in relation to the time required for the transition. This study addresses that gap by analysing the experiences of European frontrunners in landfill reduction.

Given the urgent need to drastically reduce GHG emissions and varying time required for transitions, this study aimed to assess whether MSWI or recycling offers a faster and more robust solution for GHG reduction in MSW management. To achieve this, the study evaluated the speed of transition from landfilling to MSWI and recycling in countries that have rapidly reduced landfilling in recent years. Using life cycle assessment (LCA), the speed of GHG emission reduction was calculated in selected cases, and the robustness of the results was tested through scenario analysis. The study findings demonstrated the inherent uncertainty in assessing the consequences of landfill diversion on GHG emissions of MSW management, emphasizing the need for rigorous uncertainty assessment and the identification of robust solutions for emission reduction. The study pointed to potential scenarios where transitioning to MSWI could reduce GHG emissions more rapidly than transitioning to recycling.

## Methodology

**Figure 1.**
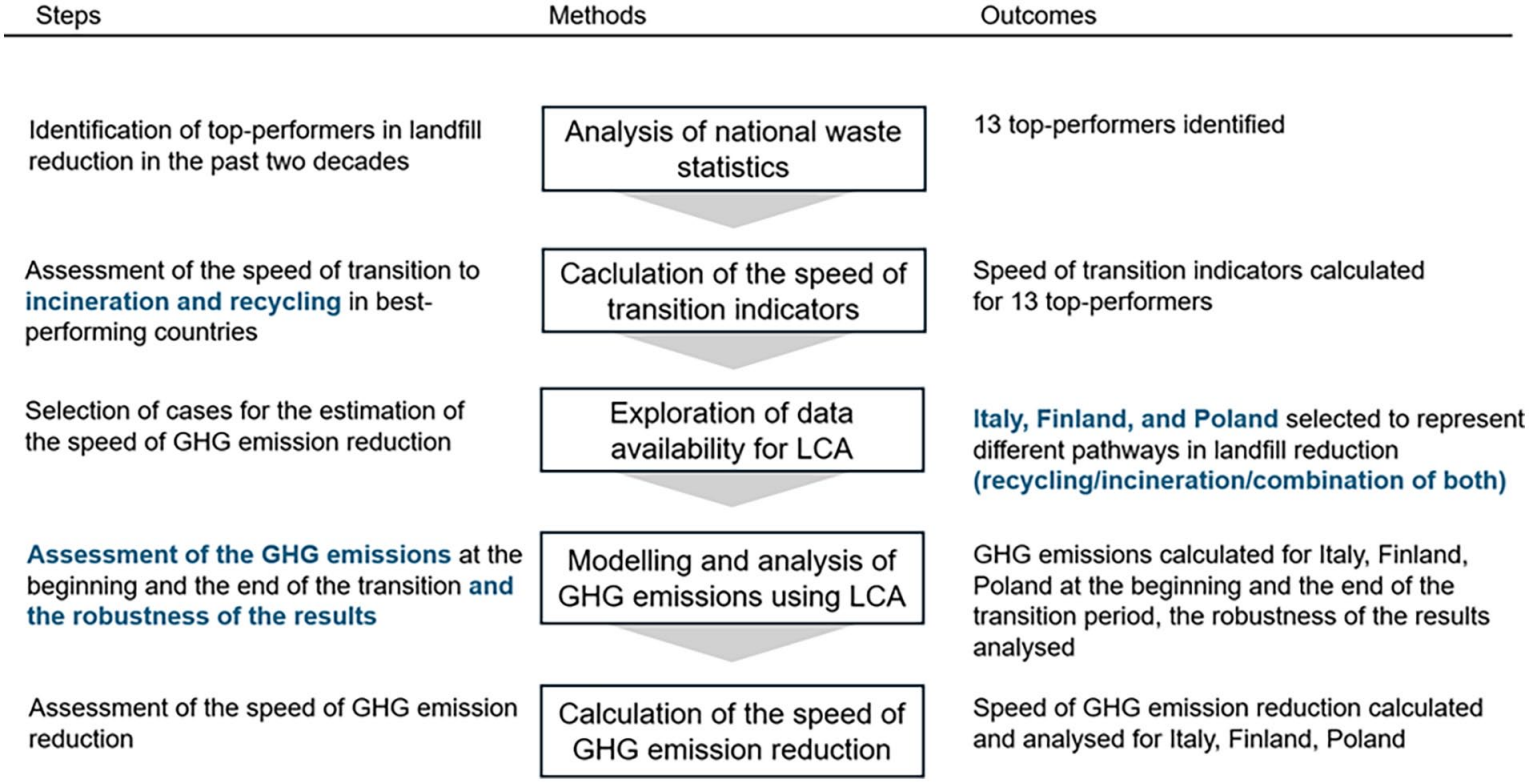
Summary of the steps, methods and outcomes of the study.

No previously published studies have retrospectively examined the performance of different GHG emission reduction strategies in relation to the time required for emission reductions to occur. Thus, the following methodology was developed. Firstly, MSW statistics of 13 countries – Lithuania, Latvia, Slovenia, Slovakia, Croatia, Ireland, Austria, Norway, United Kingdom, Finland, Poland, Italy and China – that performed well in the transition from MSW landfilling to recycling and/or MSWI in a short period of time were analysed. The speed of transition was quantitatively assessed using mathematical equations that consider the percentage changes in waste incineration, recycling and landfilling relative to the period of time in which the shift from landfilling to recycling or MSWI happened (hereinafter referred to as the transition period). The equations are defined in subsection ‘Speed of transition indicators’. Calculated indicators allowed evaluation and comparison of the progress of each country in diverting waste from landfills. To assess GHG emission reduction resulting from landfill diversion, LCA was conducted for MSW management in three cases: Finland, Italy and Poland, all at the start and end point of their transition periods. These three cases were chosen based on availability of waste data, particularly data on amounts of source separated waste for different fractions of MSW. Transition periods were also corrected where necessary based on availability of data. The cases of Finland, Italy and Poland may exemplify different directions taken in landfill diversion, as Finland mostly developed MSWI, Italy focused on recycling, whereas Poland developed MSWI and recycling in similar proportions. Goal and scope of LCA and its inventory are provided in subsection ‘Life cycle assessment’. Then, the speed of GHG emission reduction was defined as an indicator to compare selected cases with regard to the speed of reducing emissions in MSW management (subsection ‘Speed of GHG emission reduction’). Lastly, scenario analysis was done to investigate how the results are affected by modelling choices (subsection ‘Scenario analysis’). Summary of the steps, methods and outcomes of this study is shown in Figure 1.

### Speed of transition indicators

To calculate and compare the speed of transition to recycling and MSWI in 13 selected countries, the following equations were applied. Equation (1) was used to quantify the speed of transition from landfilling to incineration of MSW with energy recovery (*speed of transition to incineration*) by measuring the change in the percentage of incinerated MSW over the transition period relative to the duration of the transition period (%-point year^−1^). In a similar manner, equation (2) was used to quantify the speed of transition from MSW landfilling to recycling – *speed of transition to recycling*, %-point year^−1^. Equation (3) quantified the *speed of landfill reduction* as it measures the change in the percentage of landfilled MSW over the transition period relative to the duration of the transition period (%-point year^−1^).

(1) $$\mathrm{Speed}\;\mathrm{of}\;\mathrm{transition}\;\mathrm{to}\;\mathrm{incineration}=\frac{\left(\frac{{\mathrm{MI}}_{2}}{{\mathrm{MG}}_{2}}\;-\frac{{\mathrm{MI}}_{1}}{{\mathrm{MG}}_{1}}\right)\;\times \;100\%}{X_{2}\;-\;\;X_{1}},$$

(2) $$\mathrm{Speed}\;\mathrm{of}\;\mathrm{transition}\;\mathrm{to}\;\mathrm{recycling}=\frac{\left(\frac{{\mathrm{MR}}_{2}}{{\mathrm{MG}}_{2}}\;-\;\;\frac{{\mathrm{MR}}_{1}}{{\mathrm{MG}}_{1}}\right)\;\times \;100\%}{X_{2}\;-\;X_{1}},$$

(3) $$\mathrm{Speed}\;\mathrm{of}\;\mathrm{landfill}\;\mathrm{reduction}=\frac{\left(\frac{{\mathrm{ML}}_{2}}{{\mathrm{MG}}_{2}}\;-\;\frac{{\mathrm{ML}}_{1}}{{\mathrm{MG}}_{1}}\right)\;\times \;100\%}{X_{2}\;-\;X_{1}},$$

where MG_1_ and MG_2_ – mass of MSW generated at the start ( $X_{1}$ ) and end ( $X_{2}$ ) point of transition period, respectively, (Mg year^−1^); MI_1_ and MI_2_ – mass of MSW incinerated at the start ( $X_{1}$ ) and end ( $X_{2}$ ) point of transition period (Mg year^−1^); MR_1_ and MR_2_ – mass of recycled MSW at the start ( $X_{1}$ ) and end ( $X_{2}$ ) point of transition period (Mg year^−1^); ML_1_ and ML_2_ – mass of landfilled MSW at the start ( $X_{1}$ ) and end ( $X_{2}$ ) point of transition period (Mg year^−1^).

Speed of transition indicators from equations (1) to (3) were calculated using data from national reports on waste management of respective countries. The used values and references to data sources are given in Supplemental Material, Section A.

### Life cycle assessment

#### Goal and scope

The aim of LCA in this study was to compare GHG emissions associated with MSW management in selected countries in the beginning and end of the respective transition periods. The functional unit employed in this LCA was one Mg of treated MSW. The LCA followed ISO 14040 (2006) and ISO 14044 (2006) standards. Modelling was performed using LCA for Experts (former GaBi) software from Sphera Solutions GmbH in Leinfelden-Echterdingen, Germany (database version 10.7.1.28), whereas some data inputs were sourced from the ecoinvent database (version 3.10) for the ‘allocation, cut-off by classification’ system model. GWP was calculated using CML2001 – August 2016 method over a 100-year period, excluding biogenic carbon. Multifunctionality of waste management system was addressed by substitution.

#### System boundary

**Figure 2.**
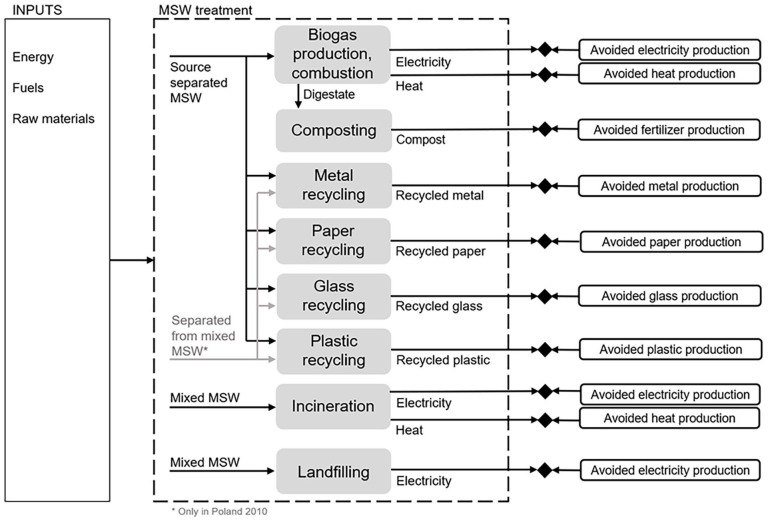
System boundary of LCA study for Finland, Italy and Poland at the start and end points of transition period. LCA: life cycle assessment.

The system boundary of MSW management system in this study was limited to include the main waste treatment processes: recycling processes, anaerobic digestion, composting, MSWI and landfilling as well as avoided production of energy and materials. Figure 2 provides a visual representation of the system boundary used. The same system boundary was used for Finland, Italy and Poland to model GHG emissions from MSW management systems at the start and end of transition period.

#### Inventory data on material flows and waste composition

Table 1 provides the amounts of MSW sent to different types of waste treatment which in total amount to one Mg of MSW. Table 1 also reports on textile, wood and bulky waste that were reported in national waste statistics to be sent to recycling, but recycling processes for those fractions were not modelled in this study due to uncertainty in required modelling assumptions and relatively small amount of those waste fractions in total amount of waste treated. LCA model of MSW management was built for 2010 and 2020 in Finland and Poland, and 2012 and 2020 in Italy due to lack of source separation statistics for Italy in 2010. The composition of mixed MSW used in the model at the beginning and end of the transition period in each country is given in Supplemental Material, Section B.

**Table 1.** Material flows in Finland, Italy and Poland used in LCA model. The values were recalculated for one Mg of MSW based on data from referenced data sources.

Waste stream	Finland 2010 (Statistics Finland, 2021)	Finland 2020 (Statistics Finland, 2021)	Italy 2012 (ISPRA, 2015)	Italy 2020 (ISPRA, 2021)	Poland 2010 (GUS, 2011)	Poland 2020 (GUS, 2021)
Separately collected waste sent to material recovery						
Biowaste	0.098	0.135	0.167	0.260	0.019	0.155
Paper and cardboard	0.111	0.132	0.105	0.127	0.017	0.048
Plastic	0.004	0.025	0.031	0.057	0.012	0.047
Glass	0.025	0.022	0.055	0.081	0.021	0.070
Metals^a^	0.005	0.035	0.009	0.013	0.002	0.005
Textile^a^			0.003	0.005	0.004	0.003
Wood^b^	0.008	0.028	0.021	0.032		
Bulky waste^b^	0.075	0.039	0.010	0.010	0.010	0.068
Waste separated from mixed MSW and sent to material recovery^c^						
Biowaste	–	–	–	–	0.061	–
Other fractions^d^			–	–	0.110	–
To material recovery, total	0.326	0.419	0.401	0.585	0.256	0.387
To MSWI	0.221	0.579	0.192	0.204	0.010	0.215
To landfill	0.453	0.005	0.407	0.211	0.734	0.398

LCA: life cycle assessment; MSW: municipal solid waste; MSWI: municipal solid waste incineration.

a Shares of metal in the composition of mixed waste was further specified using data from (KIVO, 2021) and (Andreasi Bassi et al., 2017).

b Recycling of these waste fractions was not modelled due to uncertainty in required modelling assumptions.

c This category was specified only in Polish statistics.

d Because data on specific waste fractions were not provided, the amount was assumed to contain 57% of plastics, 40% of paper and 3% of metals based on Den Boer (2017).

#### Inventory for energy use and energy recovery

In the initial LCA model, electricity and heat substitution and consumption was modelled using average data specific to each country and year. Average mixes were built using data on electricity and heat generation in all cases. Assumed energy mixes were reported in detail in Supplemental Material, Section C. In electricity mixes, the percentage of fossil energy sources change from 93% to 79% in Poland, from 74% to 58% in Italy and from 41% to 14% in Finland over the transition period. Generally, similar trends occur in heat mixes. Using average data follows an attributional approach in LCA, which aims to evaluate environmental impacts by accounting for relevant physical flows to and from the system throughout its life cycle (Ekvall and Weidema, 2004). Other modelling choices are tested in scenario analysis.

#### Inventory for material recovery processes

Prior to recycling, separately collected recyclables were assumed to be sorted to remove impurities. Sorting efficiencies applied in the model can be found in Supplemental Material, Section D. For recyclables separated from mixed MSW (in case of Poland, 2010), sorting efficiencies were not used in the model as the relevant waste amount from Table 1 was assumed to be sorting output.

The LCA model included recycling of plastic, glass, metals, paper and treatment of biowaste. Metals recycling was specified into recycling of aluminium and steel; recycling of all the separated plastic was modelled as recycling of PET, recycling of paper and cardboard was modelled as recycling of paper. It was assumed that wastepaper is used to produce newsprint paper. For secondary and primary production of PET, paper and aluminium, data from the ecoinvent 3.10 database were used. Glass recycling was modelled using data from Gaines and Mintz (1994) and Landi et al. (2019), whereas primary production of glass was based on data from LCA for Experts 10.7.1.28. Full data on modelling of recycling, including substitution ratios that were used to model replacement of virgin materials, are given in Supplemental Material, Section D.

In all cases, the full amount of collected biowaste was assumed to be used in biogas production, followed by composting of digestate. Dry anaerobic digestion process was modelled based on data from Jensen et al. (2017) with 85 kWh of electricity and 23 kWh of heat per Mg of treated biowaste sold to the grid and heating network. Open windrow composting process from LCA for Experts 10.7.1.28 database was used for treatment of digestate. Produced compost was assumed to substitute mineral fertilizers. Replacement of N, P, and K fertilizers was modelled based on Boldrin et al. (2009), and GHG emission from the production of replaced materials were based on Hansen et al. (2006)

#### Inventory for waste incineration

To model MSWI, processes of incineration of paper, biodegradable waste, plastic, wood, textile, ferrous metals and glass/inert material from the LCA for Experts 10.7.1.28 database were used as a basis (processes are reported in Supplemental Material, Section E). Recovery of electricity and heat was assumed in all cases. Energy outputs of LCA for Experts processes were modified according to energy recovery efficiencies specific to Finland, Italy and Poland. Electricity and heat production efficiencies used in the model equal to 23% and 17% in Italy (ISPRA, 2022), 14% and 49% in Poland (Cyranka et al., 2016) and 11% and 73% in Finland (CEWEP, 2020), respectively. Possible change of energy recovery rates over years was not considered due to incompleteness of available data. Substitution of heat and electricity was modelled based on country-specific heat and electricity mix as described in subsection ‘Inventory for energy use and energy recovery’.

#### Inventory for landfilling

Generation of methane resulting from decomposition of MSW was modelled based on IPCC default model (Jensen and Pipatti, 2000) for paper, wood, biowaste and textiles, supplemented by data from Lee et al. (2017). The inventory for landfilling included the use of electricity and diesel for landfill operation and the related emissions. LFG collection rate was assumed to be 37% in Finland, 48% in Italy and 12% in Poland, based on estimations for methane recovery on the national level from Oonk (2012) and expert evaluation. Combustion and flaring of LFG was modelled based on literature data. The energy produced from LFG combustion substituted electricity in all cases. The full inventory on landfilling is available in Supplemental Material, Section F.

### Speed of GHG emission reduction

Equation (4) was used to calculate the reduction in GHG emissions in MSW management system during the transition period, measured in kg CO_2_-eq. (Mg MSW treated·year)^−1^:

(4) $$Speed\;of\;emission\;reduction=\frac{E_{1}-E_{2}}{X_{2}-X_{1}},$$

where $E_{1},E_{2}$ are total amounts of GHG emissions from MSWI, recycling and landfilling per Mg of waste treated (kg CO_2_-eq. Mg MSW treated^−1^) at the start ( $X_{1}$ ) and end point ( $X_{2}$ ) of transition period, respectively.

### Scenario analysis

In waste LCA, energy modelling affects climate impacts of waste management systems significantly, especially when energy recovery technologies are in the focus of analysis. Using marginal data instead of average is recommended in such cases, although identification of marginal data can be complex and associated with uncertainties (Fruergaard et al., 2009). Use of marginal data generally complies with the consequential approach in LCA, which is relevant when studying transitions, since consequential LCA aims to assess the consequences of the changes in the system. The principles of marginal technology identification were described by Weidema et al. (1999). According to Weidema et al. (1999), marginal technologies, which are defined as the actual technologies (for instance, electricity production technologies) that are affected by changes in the system, depend on market trends (following the same example, on an electricity market). When the market is growing or decreasing slower than the average capital replacement rate, the technologies used to meet the increasing demand are considered marginal. In a declining market, the technologies being phased out are considered marginal.

However, even when analysing past changes in energy systems, marginal energy technologies may be identified differently depending on the timeframe used for identifying marginal data, as fluctuations in energy generation can blur the distinction between growth and decline trends. Besides, fluctuating energy production can couple with the simultaneous decrease in fossil energy use and the increase in renewable energy use. One example of these issues is electricity generation in Finland (see IEA, 2023b). Between 2000 and 2020, the electricity market was relatively stable, thus, according to Weidema et al. (1999), the energy sources which increased their shares in the mix (mostly wind) would be used as marginal. But between 2010 and 2020, the electricity market decreased, and thus, energy sources that decreased their shares (mostly coal) would be marginal energy technologies. Additionally, phasing out of coal and natural gas in the electricity mix was happening at the same time as wind energy was expanding. To address these uncertainties, three cases for marginal energy data were tested in the scenario analysis: (a) marginal mix, which was identified fully following principles from Weidema et al. (1999) and Consequential LCA (2021), (b) single marginal energy sources that decreased their shares in the national energy mixes the most and (c) single marginal energy sources that increased their shares in the national energy mixes the most. For this study, marginal energy data were identified based on the heat and electricity generation statistics provided by IEA for 2005–2020 for each country and were assumed to be the same at the beginning and the end of the transition periods. The resulting marginal data and more detail on what cases (a), (b) and (c) represent are given in Table 2.

**Table 2.** Marginal energy sources identified for Finland, Italy and Poland based on IEA (IEA, 2023a, 2023b, 2023c) for the scenario analysis.

Energy type modelled	Case (a): Energy sources identified as marginal following Weidema et al. (1999) and Consequential LCA (2021) fully, marginal mix	Case (b): Energy source that increased their presence in the energy mix the most, single marginal	Case (c): Energy source that decreased their presence in the energy mix the most, single marginal
	– represents moderate conditions – depends on whether the market increases or decreases strictly	– represents more specific and, in most cases, also rather extreme conditions (the most fossil/fossil-free energy) – does not depend on whether the market increases or decreases to avoid uncertainty of fluctuating markets but captures the main trends	
Finland			
Electricity	Wind – 40% Solar PV – 5% Natural gas – 37% Biofuels – 18%	Wind	Coal
Heat	Biofuels – 100%	Biofuels	Coal
Italy			
Electricity	Wind – 16% Solar PV – 21% Natural gas – 50% Biofuels – 13%	Natural gas	Oil
Heat	Natural gas – 73% Biofuels – 27%	Natural gas	Oil
Poland			
Electricity	Wind – 36% Hydro – 18% Nuclear – 25% Biofuels – 21%	Wind	Coal
Heat	Coal – 100%	Natural gas	Coal

LCA: life cycle assessment.

Furthermore, LFG collection rate is an important parameter in waste LCAs that can bring significant uncertainty. In the scenario analysis, because country-specific uncertainty ranges were challenging to estimate, 5%–86% range for LFG collection rate was tested in addition to selected country-specific values based on Duan et al. (2022) and Oonk (2012).

## Results

### Speed of transition indicators’ results

Table 3 presents the results of quantifying the speed of transition to recycling, incineration and landfill reduction for 13 countries that excelled in diverting MSW from landfills. Lithuania, Ireland, Slovenia and Finland performed the best in overall landfill diversion. Lithuania, Slovenia and Slovakia showed the fastest transition to recycling, with annual increases of 4.02, 3.70 and 3.31 percentage points, respectively. Ireland, Finland, Norway and China were the quickest to develop MSWI, with annual increases of 5.26, 3.60, 3.70 and 3.56 percentage points, respectively. Among the countries selected for further investigation – Finland, Italy and Poland – only Finland showed notable results. Italy’s speed of transition to recycling was moderate compared to other top performers.

**Table 3.** Speed of transition indicators in countries that performed well in diverting MSW from landfills, based on equations (1)–(3).

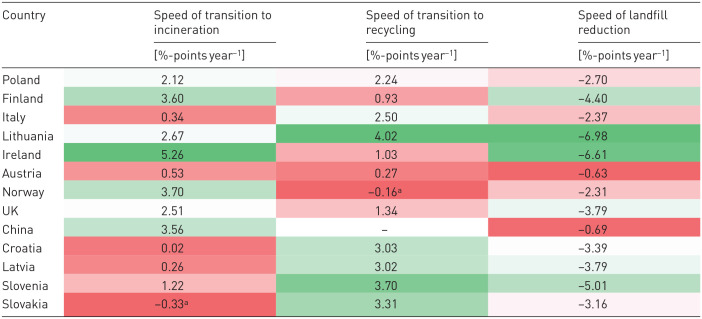			

Green colour indicates higher speed of transition, and red indicates a slower transition, with darker shades representing more extreme values and lighter shades indicating values closer to the average.

MSW: municipal solid waste.

a Negative value resulted from the increase in MSW generation.

### LCA results

**Figure 3.**
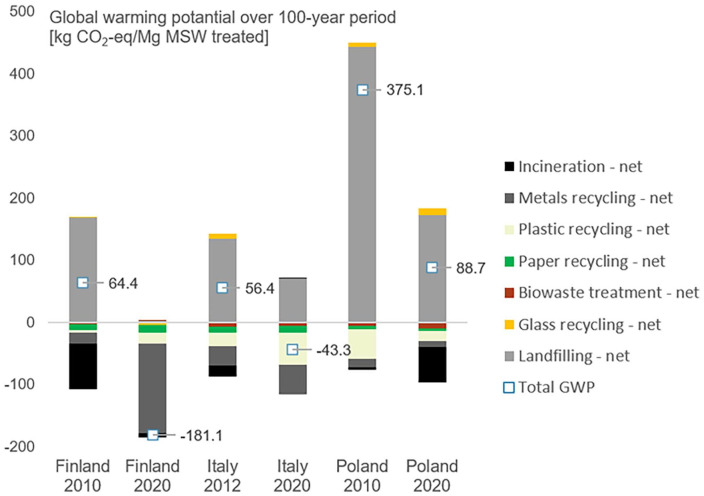
GWP of MSW management over 100-year timespan in the start and end years of the transition in Poland, Italy and Finland. GWP: global warming potential; MSW: municipal solid waste.

Figure 3 illustrates the GWP of MSW management at the start and end of the transition period in Finland, Italy and Poland. In all three countries, GHG emissions decreased, with a reduction in methane emissions from landfills playing an important role. The impact of increased MSWI and recycling had varying results. Climate impacts of MSWI was heavily affected by GHG intensity of the replaced energy mixes and their changes over the transition period, while also being a function of country-specific energy recovery efficiencies. Recycling generally showed negative GWP, with plastic and metal recycling contributing to emission savings the most. Glass recycling in Italy and Poland led to an environmental burden because virgin glass production was modelled with European average data, while energy use in recycling was based on country-specific mixes. Biowaste treatment had a negligible impact on emission reduction. Direct and avoided emissions associated with all modelled processes can be found in Table 8 of Supplemental Material, Section G.

In Finland, GHG emissions dropped from 64 kg CO_2_-eq. Mg of treated MSW^−1^ in 2010 to −181 kg CO_2_-eq. Mg of treated MSW^−1^ in 2020 due to a near elimination of emissions from landfill, and, interestingly, a significant increase in recycling, mainly metals. Despite a substantial increase in MSWI in Finland from 2010 to 2020, the benefit of MSWI decreased following a shift in the Finnish energy profile towards non-fossil sources.

In Italy, total net GWP shifted from 56 kg CO_2_-eq. Mg of treated MSW^−1^ in 2012 to −43 kg CO_2_-eq. Mg of treated MSW^−1^ in 2020. Besides a reduction in emissions from landfills, increased plastic and metal recycling contributed to GHG savings. Emission savings from MSWI in 2012 turned to a slight environmental burden in 2020 (see Supplemental Material, Section G) due to defossilization of Italian energy mix, while the amount of incinerated MSW remained almost unchanged.

Polish MSW management in 2010 had high emissions from landfills and only minor compensation from recycling benefits, resulting in a net GWP of 375 kg CO_2_-eq. per Mg of treated MSW. In 2020, net GWP dropped to 89 kg CO_2_-eq. Mg of treated MSW^−1^. Despite increased recycling, emission savings from recycling dropped in 2020, largely due to the high amount of plastic recycling modelled in 2010 based on waste statistics and assumptions (see subsections ‘Inventory data on material flows and waste composition’ and ‘Inventory for material recovery processes’). The impact of MSWI in Poland shifted from slight environmental savings in 2010 to significant savings in 2020, mainly due to increased MSWI capacity, whereas Polish energy mix remained largely fossil-based.

### Scenario analysis results

**Figure 4.**
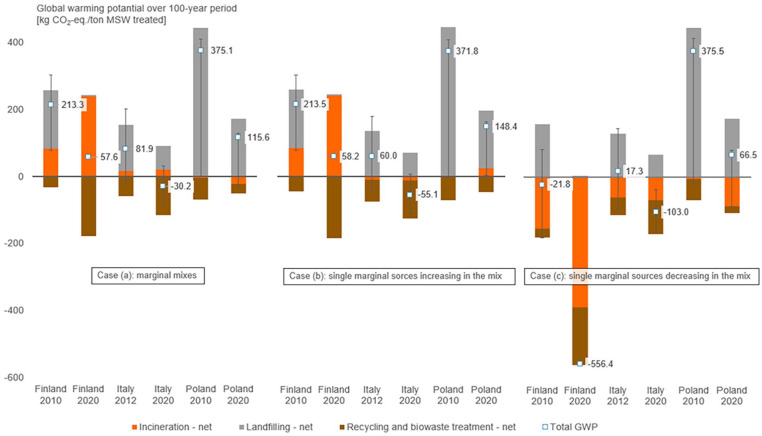
Results of scenario analysis: GWP of MSW management in the start and end years of the transition in Poland, Italy and Finland. In case (a), energy in the system was modelled as a marginal mix (often moderately carbon-intensive mixes); in case (b), energy was modelled as a single marginal energy source that increased its share in the energy mix the most (often renewable sources) and in case (c), recovered energy substitutes a single marginal energy source that decreased its share in the energy mix the most (fossil energy sources). Error bars show the effect of varying LFG collection rate from 5% to 86%. GWP: global warming potential; MSW: municipal solid waste; LFG: landfill gas.

The results of the scenario analysis are presented in Figure 4 and detailed in Supplemental Material, Section G. Figure 4 shows that the choice of background energy data affected LCA results greatly. If marginal data were based on single marginal energy sources decreasing in the energy mix (case (c), fossil energy sources), increased waste incineration contributed to GHG emission reduction and MSWI-focused transition in Finland showed the largest decrease in GWP across all countries and cases modelled, from −22 kg CO_2_-eq. Mg MSW treated to −556 kg CO_2_-eq. per Mg MSW treated. In case of single marginal energy sources increasing in the energy mix (case (b), often non-fossil), GHG emissions from MSWI mostly had positive net values, meaning that increased MSWI did not provide environmental benefits. The exception was Italy, where MSWI led to small emission savings since natural gas was the energy source that increased in the Italian energy mix the most. Despite this, GWP of MSW management systems decreased over the transition period, owing to the increased environmental savings from recycling and the reduction of methane emissions at landfills. The results of using marginal mixes (case (a)) differed from those of case (b) only slightly. The differences are noticeable in Italy and Poland, where marginal mixes (case (a)) included both renewables and natural gas, whereas the single marginal energy sources (case (b)) were either natural gas or a renewable, low-carbon energy source. Variations in GWP due to possible uncertainty in the LFG collection rate, shown in Figure 4 with error bars, were significant in all cases where landfilling is present, and illustrate how GWP reduction could vary with different practices in landfilling.

### Speed of GHG emission reduction

The ranking of the speed of GHG emission reduction differed depending on the energy data assumptions used in the LCA model, as shown in Table 4. For instance, Finland demonstrated the highest speed of GHG emission reduction among the three countries if energy produced from MSWI replaced phased-out fossil-based technologies. Poland had the fastest emission reduction in the remaining cases, which can be attributed to its significantly lower efficiency in LFG collection (12%) compared to the other countries, resulting in a greater impact from diverting waste from landfills. Italy had the slowest speed of GHG emission reduction in all cases, which can be explained by a smaller decrease in landfill use compared to the other two countries and smaller impact from MSWI (either a smaller burden or smaller savings). This is because in Italy energy recovered in MSWI replaced energy sources with more moderate carbon intensity in nearly all cases (see subsection ‘Inventory for energy use and energy recovery’ and Table 2). Overall, the results for the speed of GHG emission reduction and their analysis show that the speed of emission reduction cannot be attributed solely to a specific emission reduction strategy (recycling vs MSWI). Additionally, assumptions about avoided energy production can be critical for determining both the magnitude and speed of GHG emission reduction.

**Table 4.** Speed of GHG emissions reduction during the transition period in Finland, Italy and Poland, kg CO_2_-eq. (Mg MSW treated·year)^−1^, based on GWP over 100-year timespan.

Country	Finland	Italy	Poland
Transition period	2010–2020	2012–2020	2010–2020
Based on average energy data	25	12	29
Based on marginal energy data			
Case (a) – marginal mix	16	14	26
Case (b) – single marginal increasing in the mix	16	14	22
Case (c) – single marginal decreasing in the mix	53	15	31

GHG: greenhouse gas; GWP: global warming potential; MSW: municipal solid waste.

## Discussion

Evaluation and comparison of emission reduction speed brought by transitioning to recycling and incineration faced challenges and limitations. Firstly, reliability of results may be challenged by current inaccuracy in waste statistics. Although the European Union (EU) adopted stricter criteria for calculation and reporting recycling rates (Official Journal of the European Union, 2019), 2010 and 2020 data followed the previous approach, likely overestimating actual recycling. Furthermore, a number of assumptions were made in the LCA model: paper, steel, aluminium, PET and glass were used to represent a more diverse profile of the recycled materials that exists in reality; assumptions had to be done to model recycling of waste separated from mixed MSW in Poland, 2010. Recycling and virgin material production was mostly modelled using data embodied in respective processes in LCA databases, consequently, the effect of different assumptions on energy in scenario analysis was not captured by the model fully. Additionally, in the scenario analysis, the assessment of marginal data effects was limited by using the same marginal data for both the beginning and end of the transition, whereas in reality, these could differ.

The specific results of this study are not directly generalizable to other countries, as they could differ due to variations in landfilling practices, waste composition, recycling systems, energy recovery efficiencies as well as national energy mixes and energy system transition trends. For instance, in this study, a sharp drop in GHG emissions in Finland, if fossil energy is replaced, is partly caused by high efficiency of heat recovery in MSWI, which may not be in demand in other countries. Therefore, conclusions should be drawn on a case-by-case basis, as different combinations of conditions are possible. Nevertheless, this paper also provides insights relevant for decision-making in MSW planning globally, which are discussed further.

LCA modelling in this study applied both consequential and attributional approaches by incorporating average and marginal energy data. The results obtained using average data illustrate how the environmental impacts of MSW management systems evolved over the transition period, providing static snapshots of impacts at specific points in time. The results based on marginal data attempt to represent the environmental consequences of the transitions that occurred. The latter approach thus is more appropriate to study transitions and usually recommended to support decision-making. However, this study showed how energy system transitions create uncertainties critical for identifying effective emission reduction pathways. An optimal approach to support decision-making for future MSW management then requires testing a range of marginal data, as recommended by Fruergaard et al. (2009). However, since factors beyond background energy data – LFG collection rates and likely other factors – can also significantly influence the results, efficient and robust solutions for GHG emission reduction are likely to be constrained by specific conditions. Identifying such solutions requires a comprehensive uncertainty analysis of GHG emissions that considers multiple influencing parameters and their interplay.

In the future, MSW management may involve changing waste compositions, increased energy recovery efficiencies and possibilities for recycling, but Bisinella et al. (2024) stated that defossilization of energy system may be ‘the single-most important future change affecting waste LCAs from a climate change prospective’. Many EU countries committed to reduce fossil fuels use in energy production (European Commission, 2019) and have already made progress in decarbonization. Some nations are expected to continue relying heavily on fossil fuels due to vast reserves, economic dependence on fossil fuel industries and lack of renewable energy potential. Globally, the U.S. Energy Information Administration (EIA, 2023) projects that by 2050, the majority of growing electricity demand will be met by non-fossil sources, with natural gas filling most of the remaining demand. Energy consumption in both industrial and residential sectors is projected to increase through 2050, though detailed forecasts for heat demand remain uncertain. In heat sources, the EIA also anticipates a shift to renewables by 2030, which could result in smaller emission savings from heat production in MSWI as well.

## Conclusions

This article raises discussion on the speed of landfill diversion and the potential reduction in GHG emissions, as landfilling remains the primary method for MSW treatment globally and a major source of human-related methane emissions. The speed of GHG emission reduction was assessed retrospectively in three of the top landfill-diverting countries: Finland, which primarily transitioned to incineration; Italy, which focused on recycling; and Poland, which balanced incineration and recycling. The results did not show a clear link between the speed of emission reduction and a specific landfill diversion strategy. Instead, it was influenced by landfilling practices – making Poland the fastest in emission reduction in several cases tested – and changes in the national energy sector and their reflection in LCA. Nevertheless, the results allow to suggest that in countries where growing energy demand is unlikely to be met primarily with fossil-free sources, waste incineration with energy recovery may accelerate GHG emission reductions more effectively than recycling. It should be noted, however, that while this study focused on the urgency of climate action, other factors may influence the choice of landfill diversion strategy.

Additionally, the findings of this study highlight key considerations for providing policymakers with reliable data. Firstly, a consequential approach should be applied when selecting landfill diversion strategies. Secondly, current transitions in the energy system introduce uncertainties that are critical for identifying the most effective emission reduction pathways. Addressing these uncertainties, alongside other influencing factors, may require comprehensive uncertainty analysis and identification of robust solutions.

## Supplemental Material

sj-docx-1-wmr-10.1177_0734242X251340318 – Supplemental material for Transition to recycling versus incineration in municipal solid waste management: Evaluating the speed of greenhouse gas emission reduction Supplemental material, sj-docx-1-wmr-10.1177_0734242X251340318 for Transition to recycling versus incineration in municipal solid waste management: Evaluating the speed of greenhouse gas emission reduction by Anna Zaikova, Amirmehrab Falsafi, Amirsohrab Falsafi, Mariam Abdulkareem and Mika Horttanainen in Waste Management & Research
