# Supplementary material for: Transition to recycling versus incineration in municipal solid waste management: Evaluating the speed of greenhouse gas emission reduction
Source: Waste Manag Res. 2025 Aug 4;43(12):2104–15. doi: 10.1177/0734242X251340318 (PMC12618704; doi:10.1177/0734242X251340318)
Supplement: sj-docx-1-wmr-10.1177_0734242X251340318 – Supplemental material for Transition to recycling versus incineration in municipal solid waste management: Evaluating the speed of greenhouse gas emission reduction [file sj-docx-1-wmr-10.1177_0734242X251340318.docx]

**Transition to recycling vs. energy recovery in municipal solid waste management: evaluating the speed of greenhouse gas emission reduction**

**Supplementary materials**

[1. Section A. Values used to calculate speed of transition indicators. 2](#_Toc198566342)

[2. Section B. Composition of mixed MSW. 3](#_Toc198566343)

[3. Section C. Average energy mixes. 3](#_Toc198566344)

[4. Section D. Recycling. 5](#_Toc198566345)

[5. Section E. Incineration. 6](#_Toc198566346)

[6. Section F. Landfilling. 7](#_Toc198566347)

[7. Section G. LCA results. 8](#_Toc198566348)

[References 10](#_Toc198566349)

# Section A. Values used to calculate speed of transition indicators.

The statistical data required for Equations 1-3 were collected from the databases and national waste management reports of each country.

Table 1. The amount of MSW generated, incinerated, recycled, and landfilled that is shown as X_1_ for the start point of the transition and X_2_ for the end point (China Statistical Yearbook, 2020; Eurostat, 2022). All amounts are given in Gg (thousand tons).

| Country | Transition Period | MSW  Generated  X_1_ | MSW Generated  X_2_ | MSW Incinerated  X_1_ | MSW Incinerated  X_2_ | MSW Recycled  X_1_ | MSW Recycled  X_2_ | MSW Landfilled  X_1_ | MSW Landfilled  X_2_ |
| --- | --- | --- | --- | --- | --- | --- | --- | --- | --- |
| Ireland | 2008 to 2014 | 3224 | 2619 | 82 | 893 | 1083 | 1042 | 1939 | 537 |
| Lithuania | 2010 to 2020 | 1253 | 1350 | 1 | 349 | 61 | 609 | 1079 | 220 |
| Austria | 2005 to 2020 | 4732 | 7438 | 1310 | 2652 | 2759 | 4634 | 535 | 137 |
| Poland | 2010 to 2020 | 12032 | 13117 | 39 | 2823 | 1 961 | 5 077 | 8037 | 5218 |
| Finland | 2010 to 2020 | 2519 | 3296 | 556 | 1908 | 826 | 1388 | 1136 | 30 |
| Norway | 2006 to 2013 | 2140 | 2518 | 675 | 1446 | 862 | 987 | 390 | 52 |
| UK | 2005 to 2018 | 35 212 | 30 786 | 2942 | 12615 | 9377 | 13 577 | 22569 | 4613 |
| China | 2010 to 2017 | 158048 | 215209 | 23000 | 102 000 | - | - | 95000 | 120 000 |
| Italy | 2012 to 2020 | 29 994 | 28 945 | 5529 | 5615 | 10 056 | 16 160 | 11720 | 5817 |
| Latvia | 2010 to 2020 | 680 | 909 | 0 | 24 | 64 | 360 | 617 | 480 |
| Slovenia | 2010 to 2020 | 1 024 | 225 | 9 | 134 | 225 | 607 | 571 | 69 |
| Slovakia | 2010 to 2020 | 1 719 | 2 612 | 183 | 193 | 156 | 1 102 | 1325 | 1189 |
| Croatia | 2010 to 2020 | 1 630 | 1 693 | 0 | 3 | 65 | 581 | 1537 | 1023 |

# Section B. Composition of mixed MSW.

Table 2. Composition of mixed MSW in Finland, Italy, and Poland used in LCA model in the beginning and the end of the transition period, given in percentages (%).

|  | Finland  2010  (Horttanainen et al., 2013) | Finland  2020  (KIVO, 2021) | Italy  2009 (Calabrò, 2009) | Italy  2020  (Istat, 2022) | Poland  2010  (Marcinkowski and Kowalski, 2012) | Poland  2020  (Jakubus and Stejskal, 2020) |
| --- | --- | --- | --- | --- | --- | --- |
| Biowaste | 23.9 | 32.3 | 32.0 | 35.0 | 32.5 | 37.6 |
| Paper& cardboard | 14.9 | 17.0 | 25.0 | 21.7 | 26.7 | 11.6 |
| Wood | 11.9 | 1.5 | 4.0 | 3.4 | 0.4 | 0.6 |
| Plastics | 21.4 | 16.6 | 11.5 | 12.6 | 13.2 | 16.6 |
| Glass | 2.5 | 2.3 | 8.0 | 10.0 | 9.8 | 11.5 |
| Metal ^a^ | 3.8 | 2.4 | 4.0 | 5.0 | 3.6 | 0.5 |
| Textiles | 9.5 | 6.1 | 1.5 | 3.6 | 1.2 | 0.2 |
| Other^b^ | 12.1 | 21.8 | 14.0 | 8.7 | 13.0 | 21.5 |
| ^a^ Steel and aluminium were assumed to comprise metal fraction; the same composition data (Andreasi Bassi et al., 2017; KIVO, 2021) were applied to the beginning and end of the transition period.  ^b^ Other waste fraction was considered to be inert in the model. | | | | | | |

# Section C. Average energy mixes.

Energy mixes were modelled using the shares of each energy source from tables below and corresponding processes from LCA for Experts 10.7.1.28. specific to each country.

Table 2. Shares of energy sources used in electricity mixes based on data from (IEA, 2022a, 2022b, 2022c)

|  | Coal | Natural Gas | Heavy Oil | Biofuel | Hydro power | Nuclear power | Wind power | Geothermal Energy | Solar Energy |
| --- | --- | --- | --- | --- | --- | --- | --- | --- | --- |
| Poland  2010 | 0.88 | 0.03 | 0.02 | 0.04 | 0.02 | 0.00 | 0.01 | - | - |
| Poland  2020 | 0.68 | 0.11 | 0.00 | 0.11 | 0.00 | 0.00 | 0.10 | - |  |
| Italy  2010 | 0.15 | 0.52 | 0.07 | 0.03 | 0.18 | 0.00 | 0.03 | 0.02 | - |
| Italy  2020 | 0.05 | 0.49 | 0.04 | 0.06 | 0.18 | 0.00 | 0.07 | 0.02 | 0.09 |
| Finland 2010 | 0.27 | 0.14 | 0.00 | 0.14 | 0.16 | 0.29 | 0.00 | - | - |
| Finland 2020 | 0.08 | 0.06 | 0.00 | 0.16 | 0.23 | 0.34 | 0.12 | - | - |

Table 3. Shares of energy sources used in heat mixes based on data from (IEA, 2022b, 2022a)

|  | Coal | Natural Gas | Heavy Oil | Solid biomass |
| --- | --- | --- | --- | --- |
| Poland  2010 | 0.89 | 0.06 | 0.02 | 0.03 |
| Poland  2020 | 0.81 | 0.11 | 0.01 | 0.07 |
| Italy  2010 | 0.01 | 0.65 | 0.31 | 0.04 |
| Italy  2020 | 0.02 | 0.69 | 0.15 | 0.16 |
| Finland  2010 | 0.21 | 0.31 | 0.07 | 0.21 |
| Finland  2020 | 0.14 | 0.15 | 0.02 | 0.53 |

# Section D. Recycling.

Table 4. Data used in modelling of sorting and recycling of paper, plastic, glass, and steel.

| Process/parameter | Value | Unit | Reference |
| --- | --- | --- | --- |
| **Paper and cardboard** | | | |
| Separation rate | 0.97 | share | (Rigamonti et al., 2010) |
| Recycling process | paper production, newsprint, recycled (Europe without Switzerland) | | ecoinvent 3.10 |
| Substituted process | paper production, newsprint, virgin (RER) | | ecoinvent 3.10 |
| Substitution ratio | 0.83 | kg _virgin paper_ /kg _recycled paper_ | (Gala et al., 2015) |
| **PET recycling** | | | |
| Separation rate | 0.67 | share | (Rigamonti et al., 2010) |
| Recycling process | Polyethylene terephthalate production, granulate, bottle grade, recycled (Switzerland) | | ecoinvent 3.10 |
| Substituted process | DE: Polyethylene terephthalate bottle grade granulate (PET) via PTA ts | | ecoinvent 3.10 |
| Substitution ratio | 0.81 | kg _virgin PET_ /kg _recycled PET_ | (Rigamonti et al., 2010) |
| **Steel recycling** | | |  |
| Separation rate | 0.91 | share | (Rigamonti et al., 2010) |
| Credit for recycled steel | GLO: Credit for recycling of steel scrap ts <Mfg> | | LCA for Experts 10.7.1.28 |
| **Aluminum recycling** |  | |  |
| Separation rate | 0.91 | share | (Rigamonti et al., 2010) |
| Recycling process | aluminium production, primary, ingot | | ecoinvent 3.10 |
| Substituted process | treatment of aluminium scrap, post-consumer, prepared for recycling, at remelter | | ecoinvent 3.10 |
| Substitution ratio | 1 | kg _virgin Al_ /kg _recycled Al_ | (Rigamonti et al., 2010) |
| **Glass recycling** | | | |
| Separation rate | 0.82 | share | (Rigamonti et al., 2010) |
| Recycling process | | | |
| -water consumption | 0.2 | m^3^/Mg_cullet_ | (Landi et al., 2019) |
| -electricity consumption | 551 | kWh/Mg_cullet_ | (Gaines and Mintz, 1994) |
| -heat consumption | 6963 | MJ/Mg_cullet_ | (Gaines and Mintz, 1994) |
| Substituted process | EU-28: Container glass ts | | LCA for Experts 10.7.1.28 |
| Substitution ratio | 1 | kg _virgin glass_ /kg _recycled glass_ | (Rigamonti et al., 2010) |

# Section E. Incineration.

Table 5. Processes used to model waste incineration.

| Waste fraction in LCA model | Process used in the model (LCA for Experts 10.7.1.28) |
| --- | --- |
| Paper and cardboard | EU-28: Waste incineration of paper fraction in MSW ELCD/CEWEP |
| Glass | EU-28: Waste incineration of glass/inert material ELCD/CEWEP |
| Biowaste | EU-28: Waste incineration of biodegradable waste fraction in MSW ELCD/CEWEP |
| Plastics | EU-28: Waste incineration of plastics fraction in MSW ELCD/CEWEP |
| Metal | EU-28: Waste incineration of ferro metals ELCD/CEWEP |
| Wood | EU-28: Waste incineration of wood products ELCD/CEWEP |
| Textile | EU-28: Waste incineration of textile fraction in MSW ELCD/CEWEP |

# Section F. Landfilling.

Methane generation (kgCH_4_/kg_waste_) of waste decomposition is calculated based IPCC default model (Jensen and Pipatti, 2000):

$$L_{0}=DOC\times{DOC}_{f}\times MCF\times F\times\frac{16}{12},$$

where $DOC$ – degradable organic carbon, kg_C_/kg_waste_,

${DOC}_{f}$ – share of degradable organic carbon degraded,

$MCF$ – CH_4_ correction factor, share,

$F$ – a fraction of CH_4_ in landfill gas, share,

$\frac{16}{12}$ – correlation between carbon and methane content.

Table 6. Initial data for calculation of the methane generation potential.

| Waste fraction | DOC, kg_C_/kg_waste_ | DOC_f_, share | L, kg_CH4_/kg_waste_ |
| --- | --- | --- | --- |
| Paper | 0.4 | 0.37 | 0.059 |
| Wood | 0.43 | 0.21 | 0.036 |
| Food | 0.15 | 0.64 | 0.038 |
| Textile | 0.24 | 0.50 | 0.048 |
| Reference | (Jensen and Pipatti, 2000) | (Lee et al., 2017) | Calculated |

Table 7. LCI data on landfilling.

| Parameter | Value | Unit | Reference |
| --- | --- | --- | --- |
| CH_4_ oxidation factor | 0.1 | share | (Jensen and Pipatti, 2000) |
| CH_4_ correction factor | 0.6 | share | (Jensen and Pipatti, 2000) |
| CH_4_ fraction in LFG | 0.5 | share | (Jensen and Pipatti, 2000) |
| Diesel consumption | 2 | dm^3^/Mg_MSW_ | (Manfredi et al., 2009) |
| Electricity consumption ^a^ | | | |
| Finland | 4.5 | kWh/Mg_MSW_ | (Manfredi et al., 2009) |
| Italy | 6.5 | kWh/Mg_MSW_ | (Manfredi et al., 2009) |
| Poland | 6.5 | kWh/Mg_MSW_ | (Manfredi et al., 2009) |
| Emissions from diesel combustion (a bulldozer) | | | |
| CO_2_-eq. (CO_2_, CH_4_, N_2_O) | 2674 | g/ dm^3^_diesel_ | (VTT, 2016) |
| LFG collection rate | | | |
| Finland | 37% | % LFG generated | (Oonk, 2012) |
| Italy | 48% | % LFG generated | (Oonk, 2012) |
| Poland | 12% | % LFG generated | (Oonk, 2012) |
| LFG to flaring | | | |
| Finland | 25 | % LFG collected | (Niskanen et al., 2009) |
| Italy | 50 | % LFG collected | (Andreasi Bassi et al., 2017) |
| Poland | 70 | % LFG collected | (Andreasi Bassi et al., 2017) |
| Gas engine electric efficiency | 37 | % | (Olesen et al., 2014) |
| Efficiency of flaring | 99 | % | (Liikanen et al., 2018) |
| Emissions from flare device | | | |
| CH_4_ | 1 | % CH_4_ input | (Bacchi et al., 2018) |
| ^a^ Electricity consumption value was chosen from Manfredi et al. (2009) assuming ‘engineered landfill for low organic waste’ as an average landfill type in Finland, and ‘conventional landfill’ as an average landfill type in Poland and Italy. | | | |

# Section G. LCA results.

Table 8. LCA results for GWP in Poland, Italy, and Finland at the beginning and the end of the transition period, kg CO_2_-eq per Mg of MSW treated, based on average energy data.

|  | Total GWP | Biowaste treatment, direct emissions | Biowaste treatment, avoided emissions | Glass recycling, direct emissions | Glass recycling, avoided emissions | Incineration, direct emissions | Incineration, avoided emissions | Landfill, direct emissions | Landfill, avoided emissions | Paper recycling, direct emissions | Paper, avoided emissions | Plastic, direct emissions | Plastic, avoided emissions | Metals recycling, net |
| --- | --- | --- | --- | --- | --- | --- | --- | --- | --- | --- | --- | --- | --- | --- |
| Finland 2010 | 64.44 | 5.38 | -7.58 | 17.03 | -14.87 | 87.26 | -160.13 | 174.91 | -5.98 | 111.05 | -121.20 | 2.36 | -5.31 | -18.48 |
| Finland 2020 | -181.12 | 6.14 | -5.86 | 8.90 | -13.21 | 250.19 | -256.86 | 3.77 | -0.05 | 132.87 | -145.01 | 14.24 | -32.09 | -144.16 |
| Italy 2012 | 56.39 | 9.19 | -15.36 | 41.65 | -32.86 | 56.36 | -74.96 | 142.05 | -7.49 | 104.90 | -114.49 | 17.22 | -38.80 | -31.04 |
| Italy 2020 | -43.32 | 11.28 | -16.10 | 35.46 | -34.71 | 66.16 | -65.82 | 73.57 | -2.81 | 126.65 | -138.23 | 41.07 | -92.54 | -47.31 |
| Poland 2010 | 375.09 | 2.52 | -7.10 | 22.46 | -15.58 | 3.40 | -7.76 | 447.83 | -3.83 | 62.15 | -67.83 | 87.66 | -135.16 | -13.67 |
| Poland 2020 | 88.68 | 4.16 | -13.88 | 52.53 | -41.03 | 94.77 | -151.13 | 174.50 | -1.23 | 38.56 | -42.08 | 31.93 | -48.19 | -10.24 |

Table 9. LCA results for GWP in Poland, Italy, and Finland at the beginning and the end of the transition period, kg CO_2_-eq per Mg of MSW treated, based on marginal energy data, case (a).

|  | Total GWP | Biowaste treatment, direct emissions | Biowaste treatment, avoided emissions | Glass recycling, direct emissions | Glass recycling, avoided emissions | Incineration, direct emissions | Incineration, avoided emissions | Landfill, direct emissions | Landfill, avoided emissions | Paper recycling, direct emissions | Paper, avoided emissions | Plastic, direct emissions | Plastic, avoided emissions | Metals recycling, net |
| --- | --- | --- | --- | --- | --- | --- | --- | --- | --- | --- | --- | --- | --- | --- |
| Finland 2010 | 213.26 | 5.38 | -4.04 | 0.79 | -14.87 | 87.26 | -4.19 | 174.72 | -0.21 | 111.05 | -121.20 | 2.36 | -5.31 | -18.48 |
| Finland 2020 | 57.64 | 6.14 | -4.60 | 0.70 | -13.21 | 250.19 | -11.15 | 3.73 | 0.00 | 132.87 | -145.01 | 14.24 | -32.09 | -144.16 |
| Italy 2012 | 81.92 | 9.19 | -11.18 | 24.86 | -32.86 | 56.36 | -40.38 | 141.86 | -3.73 | 104.90 | -114.49 | 17.22 | -38.80 | -31.04 |
| Italy 2020 | -30.24 | 11.28 | -13.71 | 26.12 | -34.71 | 66.16 | -46.61 | 73.53 | -1.94 | 126.65 | -138.23 | 41.07 | -92.54 | -47.31 |
| Poland 2010 | 375.09 | 2.52 | -7.10 | 22.46 | -15.58 | 3.40 | -7.76 | 447.83 | -3.83 | 62.15 | -67.83 | 87.66 | -135.16 | -13.67 |
| Poland 2020 | 115.63 | 4.16 | -7.86 | 40.97 | -41.03 | 94.77 | -118.01 | 172.95 | -0.30 | 38.56 | -42.08 | 31.93 | -48.19 | -10.24 |

Table 10. LCA results for GWP in Poland, Italy, and Finland at the beginning and the end of the transition period, kg CO_2_-eq per Mg of MSW treated, based on marginal energy data, case (b).

|  | Total GWP | Biowaste treatment, direct emissions | Biowaste treatment, avoided emissions | Glass recycling, direct emissions | Glass recycling, avoided emissions | Incineration, direct emissions | Incineration, avoided emissions | Landfill, direct emissions | Landfill, avoided emissions | Paper recycling, direct emissions | Paper, avoided emissions | Plastic, direct emissions | Plastic, avoided emissions | Metals recycling, net |
| --- | --- | --- | --- | --- | --- | --- | --- | --- | --- | --- | --- | --- | --- | --- |
| Finland 2010 | 213.52 | 5.38 | -4.01 | 0.75 | -14.87 | 87.26 | -3.97 | 174.72 | -0.15 | 111.05 | -121.20 | 2.36 | -5.31 | -18.48 |
| Finland 2020 | 58.22 | 6.14 | -4.59 | 0.66 | -13.21 | 250.19 | -10.55 | 3.73 | 0.00 | 132.87 | -145.01 | 14.24 | -32.09 | -144.16 |
| Italy 2012 | 60.01 | 9.19 | -14.60 | 36.63 | -32.86 | 56.36 | -67.63 | 142.02 | -6.90 | 104.90 | -114.49 | 17.22 | -38.80 | -31.04 |
| Italy 2020 | -55.06 | 11.28 | -17.91 | 38.56 | -34.71 | 66.16 | -78.12 | 73.61 | -3.58 | 126.65 | -138.23 | 41.07 | -92.54 | -47.31 |
| Poland 2010 | 371.85 | 2.52 | -1.02 | 9.35 | -15.58 | 3.40 | -3.25 | 443.31 | -0.04 | 62.15 | -67.83 | 87.66 | -135.16 | -13.67 |
| Poland 2020 | 148.37 | 4.16 | -5.65 | 24.62 | -41.03 | 94.77 | -70.95 | 172.47 | -0.01 | 38.56 | -42.08 | 31.93 | -48.19 | -10.24 |

Table 11. LCA results for GWP in Poland, Italy, and Finland at the beginning and the end of the transition period, kg CO_2_-eq per Mg of MSW treated, based on marginal energy data, case (c).

|  | Total GWP | Biowaste treatment, direct emissions | Biowaste treatment, avoided emissions | Glass recycling, direct emissions | Glass recycling, avoided emissions | Incineration, direct emissions | Incineration, avoided emissions | Landfill, direct emissions | Landfill, avoided emissions | Paper recycling, direct emissions | Paper, avoided emissions | Plastic, direct emissions | Plastic, avoided emissions | Metals recycling, net |
| --- | --- | --- | --- | --- | --- | --- | --- | --- | --- | --- | --- | --- | --- | --- |
| Finland 2010 | -21.83 | 5.38 | -13.55 | 28.15 | -14.87 | 87.26 | -240.20 | 175.30 | -17.72 | 111.05 | -121.20 | 2.36 | -5.31 | -18.48 |
| Finland 2020 | -556.36 | 6.14 | -15.48 | 25.01 | -13.21 | 250.19 | -638.50 | 4.02 | -0.38 | 132.87 | -145.01 | 14.24 | -32.09 | -144.16 |
| Italy 2012 | 17.30 | 9.19 | -20.96 | 54.63 | -32.86 | 56.36 | -116.22 | 142.33 | -12.97 | 104.90 | -114.49 | 17.22 | -38.80 | -31.04 |
| Italy 2020 | -103.04 | 11.28 | -25.71 | 57.57 | -34.71 | 66.16 | -134.32 | 73.77 | -6.72 | 126.65 | -138.23 | 41.07 | -92.54 | -47.31 |
| Poland 2010 | 375.55 | 2.52 | -7.69 | 23.94 | -15.58 | 3.40 | -8.27 | 448.25 | -4.18 | 62.15 | -67.83 | 87.66 | -135.16 | -13.67 |
| Poland 2020 | 66.53 | 4.16 | -16.62 | 63.03 | -41.03 | 94.77 | -181.31 | 175.17 | -1.63 | 38.56 | -42.08 | 31.93 | -48.19 | -10.24 |

# References

Andreasi Bassi S, Christensen TH and Damgaard A (2017) Environmental performance of household waste management in Europe - An example of 7 countries. *Waste Management* 69. Pergamon: 545–557.

Bacchi D, Bacci R, Ferrara G, et al. (2018) Life Cycle Assessment (LCA) of landfill gas management: Comparison between conventional technologies and microbial oxidation systems. *Energy Procedia* 148. Elsevier B.V.: 1066–1073.

Calabrò PS (2009) Greenhouse gases emission from municipal waste management: The role of separate collection. *Waste Management* 29(7). Pergamon: 2178–2187.

China Statistical Yearbook 2020 | GHDx (2020). Available at: https://ghdx.healthdata.org/record/china-statistical-yearbook-2020 (accessed 11 November 2024).

Gaines LL and Mintz MM (1994) Energy Implications of Glass-Container Recycling. Available at: https://www.nrel.gov/docs/legosti/old/5703.pdf. *Argonne National Organization* 81(1): 65–6, 68, 2.

Gala AB, Raugei M and Fullana-i-Palmer P (2015) Introducing a new method for calculating the environmental credits of end-of-life material recovery in attributional LCA. *International Journal of Life Cycle Assessment* 20(5). Springer Verlag: 645–654.

Horttanainen M, Teirasvuo N, Kapustina V, et al. (2013) The composition, heating value and renewable share of the energy content of mixed municipal solid waste in Finland. *Waste Management* 33(12). Pergamon: 2680–2686.

IEA (2022a) Energy Statistics Data Browser – Data Tools - IEA. Available at: https://www.iea.org/data-and-statistics/data-tools/energy-statistics-data-browser?country=POL&fuel=Electricity%20and%20heat&indicator=HeatGenByFuel (accessed 11 November 2024).

IEA (2022b) Energy Statistics Data Browser – Data Tools - IEA. Available at: https://www.iea.org/data-and-statistics/data-tools/energy-statistics-data-browser?country=WORLD&fuel=Energy%20supply&indicator=TESbySource (accessed 11 November 2024).

IEA (2022c) Energy Statistics Data Browser – Data Tools - IEA. Available at: https://www.iea.org/data-and-statistics/data-tools/energy-statistics-data-browser?country=ITA&fuel=Electricity%20and%20heat&indicator=ElecIndex (accessed 11 November 2024).

Istat (2022) *Environment and economy: main indicators – Years 2018-2020 – Istat*. Available at: https://www.istat.it/en/press-release/environmente-and-economy-main-indicators-years-208-2020/ (accessed 11 November 2024).

Jakubus M and Stejskal B (2020) Municipal solid waste management systems in Poland and the Czech Republic. A comparative study. *Environment Protection Engineering* 46(3). Wrocław University of Science and Technology: 61–78.

Jensen JEF and Pipatti R (2000) Waste. In: Penman J, Kruger D, Galbally I, et al. (eds) *Good Practice Guidance and Uncertainty Management in National Greenhouse Gas Inventories*, pp. 419–439. Available at: http://www.ipcc-nggip.iges.or.jp/public/gp/bgp/5_1_CH4_Solid_Waste.pdf.

KIVO (2021) Composition data bank [Koostumustietopankki]. Available at: https://kivo.fi/ymmarramme/koostumustietopankki/ (accessed 17 January 2025).

Landi D, Germani M and Marconi M (2019) Analyzing the environmental sustainability of glass bottles reuse in an Italian wine consortium. In: *Procedia CIRP*, 1 January 2019, pp. 399–404. Elsevier B.V.

Lee U, Han J and Wang M (2017) Evaluation of landfill gas emissions from municipal solid waste landfills for the life-cycle analysis of waste-to-energy pathways. *Journal of Cleaner Production* 166: 335–342.

Liikanen M, Havukainen J, Viana E, et al. (2018) Steps towards more environmentally sustainable municipal solid waste management – A life cycle assessment study of São Paulo, Brazil. *Journal of Cleaner Production* 196. Elsevier Ltd: 150–162.

Manfredi S, Tonini D, Christensen TH, et al. (2009) Landfilling of waste: accounting of greenhouse gases and global warming contributions. *Waste Management & Research* 27: 825–836.

Marcinkowski A and Kowalski AM (2012) The problem of preparation the food packaging waste for recycling in Poland. *Resources, Conservation and Recycling* 69. Elsevier: 10–16.

Niskanen A, Manfredi S, Christensen TH, et al. (2009) Environmental assessment of Ämmässuo Landfill (Finland) by means of LCA-modelling (EASEWASTE). *https://doi-org.ezproxy.cc.lut.fi/10.1177/0734242X08096976* 27(5). SAGE PublicationsSage UK: London, England: 542–550.

Olesen AO, Urup ; and Damgaard A (2014) *Landfilling in EASETECH: Data collection and modelling of the landfill modules in EASETECH*. *Downloaded from orbit.dtu.dk on*. Kgs. Lyngby: Technical University of Denmark. Available at: https://orbit.dtu.dk/en/publications/landfilling-in-easetech-data-collection-and-modelling-of-the-land (accessed 21 January 2025).

Oonk H (2012) Efficiency of landfill gas collection for methane emission reduction. *Greenhouse Gas Measurement and Management* 2(2–3). TF: 129–145.

Rigamonti L, Grosso M and Giugliano M (2010) Life cycle assessment of sub-units composing a MSW management system. *Journal of Cleaner Production* 18(16–17): 1652–1662.

Statistics | Eurostat (2022). Available at: https://ec.europa.eu/eurostat/databrowser/view/env_wasmun/default/table?lang=en (accessed 11 November 2024).

Tilastokeskus (2023) Production of district heat by Year, Production of district heat, GWh and Information. PxWeb. Available at: https://statfin.stat.fi/PxWeb/pxweb/en/StatFin/StatFin__salatuo/statfin_salatuo_pxt_12b7.px/ (accessed 11 November 2024).

Vogt R, Derreza-Greeven C, Giegrich J, et al. (2015) *The Climate Change Mitigation Potential of the Waste Sector*. Heidelberg, Germany. Available at: http://www.umweltbundesamt.de/publikationen/the-climate-change-mitigation-potential-of-the (accessed 20 January 2025).

VTT (2016) Transport and mobility impact assessments | VTT. Available at: https://www.vttresearch.com/fi/palvelut/liikenteen-vaikutusten-ennakointi (accessed 11 November 2024).
